# Cooperative functions of manganese and thiol redox system against oxidative stress in human spermatozoa

**DOI:** 10.4103/0974-1208.57227

**Published:** 2009

**Authors:** Amrit Kaur Bansal, Anand Ravinder Jit Kaur

**Affiliations:** Department of Animal Reproduction, Gynaecology and Obstetrics, Guru Angad Dev Veterinary and Animal Sciences University, Ludhiana, Punjab University, Chandigarh, India; 1 Department of Zoology, Guru Angad Dev Veterinary and Animal Sciences University, Ludhiana, Punjab University, Chandigarh, India

**Keywords:** Human sperm, Mn^2+^, thiols

## Abstract

**AIMS:**

In this study, the effects of 0.1 mM Mn^2+^ on thiol components (total thiols [TSH], glutathione reduced [GSH], glutathione oxidized [GSSG] and redox ratio [GSH/ GSSG]) have been determined in human spermatozoa.

**SETTINGS AND DESIGN:**

The subjects of the study were healthy males having more than 75% motility and 80 × 10^6^ sperms/mL.

**MATERIALS AND METHODS:**

Fresh semen was suspended in phosphate-buffered saline (PBS) (pH 7.2) and this suspension was divided into eight equal fractions. All fractions, control (containing PBS) and experimental (treated/untreated with [ferrous ascorbate, FeAA - 200 FeSO_4_ μM, 1000 μM ascorbic acid, nicotine (0.5 mM) and FeAA + nicotine], supplemented/unsupplemented with Mn^2+^ [0.1 mM]), were incubated for 2 h at 37°C. These fractions were assessed for determining the thiol components.

**STATISTICAL ANALYSIS:**

The data were statistically analyzed by Students “*t*” test.

**RESULTS AND CONCLUSIONS:**

Ferrous ascorbate, nicotine and ferrous ascorbate + nicotine induced oxidative stress and decreased GSH and redox ratio (GSH/GSSG ratio) but increased the TSH and GSSG levels. Mn^2+^ supplementation improved TSH, GSH and redox ratio (GSH/GSSG) but decreased the GSSG level under normal and oxidative stress conditions. Thiol groups serve as defense mechanisms of sperm cells to fight against oxidative stress induced by stress inducers such as ferrous ascorbate, nicotine and their combination (ferrous ascorbate + nicotine). In addition, Mn^2+^ supplementation maintains the thiol level by reducing oxidative stress.

## INTRODUCTION

Among numerous factors that influence male fertility, oxidative stress has elicited enormous interest in the recent years.[1] Oxidative stress in the reproductive system is thought to have an effect on the fertilizing ability of sperm.[2] It occurs as a consequence of an imbalance between the production of reactive oxygen species (ROS) and their removal by antioxidant defenses.[3] Several ROS, including superoxide anion (^−^ O_2_·), hydroxyl radical (OH·) and hypochlorite radical (OHCl·) produced by both the sperm and the leukocytes contaminating the seminal fluid adversely affect the sperm motility and impair the fertilizing ability.[4,5] It has been demonstrated that men with a high level of ROS generation have a seven-fold lesser chance of initiating a pregnancy as compared with those with low ROS.[6] The combination of Fe^2+^ and ascorbate has been used to induce oxidative stress/lipid peroxidation (LPO) by increasing the level of thiobarbituric acid reactive substances such as malondialdehyde that originate from the breakdown of lipid peroxides.[7,8] Exposure to nicotine, cigarette smoking and/or polycyclic aromatic hydrocarbons is able to induce oxidative stress and cause testicular atrophy, block spermatogenesis and alter sperm morphological features.[6]

Membrane sulfhydryl (–SH) groups are the important entities of the sperm membranes and serve as defense mechanisms of the spermatozoa to fight against oxidative stress.[9,10] Decrease in motility and loss of sperm functions in unexplained male infertility can be attributed to the masking of active sulfhydryl (–SH) groups and thereby resulting in the loss of sperm functions. –SH groups can be used as tools for infertility assessment in unexplained male infertility and can be targeted for contraceptive research.[10]

Glutathione (GSH) is the major nonprotein thiol in mammalian cells and is known to have numerous biological functions.[8] The scavenging action of GSH helps to counteract the effects of oxidative stress in sperm cells, which could result in LPO of the plasmalemma, irreversible loss of motility, leakage of intracellular enzymes and damage of chromatin.[11] GSH exists in two forms: The reduced (GSH) and the oxidized (GSSG).[11] The protective action of GSH against ROS is facilitated by the interaction with its associated enzymes, such as glutathione peroxidase (GPx) and reductase (GR).[11]

An antioxidant that reduces oxidative stress and improves sperm motility could be useful in the management of male infertility.[12] Antioxidative mechanisms protect the sperms from the damage caused by free radicals.[13] The antioxidative action of Mn^2+^ on various peroxidizing systems (sperms, neurons) has been studied. It inhibits LPO produced by a free radical producing system but not LPO induced by singlet oxygen.[14] Manganese in very small amounts affects human health and its deficiency may cause symptoms such as impaired or depressed reproductive functions.[15,16] It is an essential component of several enzymes, some of which (superoxide dismutase, pseudocatalase and the photosynthetic oxygen evolving center) are involved in redox processes.[17] It has also been assigned as a chain-breaking antioxidant as it is able to quench peroxyl radicals.[18]

The present *in vitro* studies are aimed to assess the antioxidative effects of Mn^2+^ (0.1 mM) on thiol (total thiols [TSH], GSH, GSSG and redox ratio (GSH/GSSG)] components of human spermatozoa under normal and oxidative stress conditions.

## MATERIALS AND METHODS

### Semen samples

Human semen samples with more than 75% motility and 80 × 10^6^ sperm/mL were collected from healthy donors. The three subsamples of a single ejaculate from each of the five individuals were used for the analysis of each parameter. A known volume of the semen sample (*n* = 5) was taken in a centrifuge tube (prewarmed at 37°C) and centrifuged (800 × g, 5 min) and the seminal plasma was removed, the sperm pellet was washed two or three times with 0.2 M phosphate-buffered saline (PBS – 0.9% sodium chloride + 0.2 M monobasic sodium phosphate + 0.2 M dibasic sodium phosphate) (pH-7.2), re-suspended in PBS and divided into the following two sets:

**Table:** 

Sets	Tubes			
	
	1	2	3	4
I	PBS	FeAA	Nicotine	FeAA + nicotine
II	Mn^2+^	FeAA + Mn^2+^	Nicotine + Mn^2+^	FeAA + nicotine + Mn^2+^

Ferrous ascorbate (FeAA - 200 FeSO_4_ μM: 1000 μM ascorbic acid); Nicotine (0.5 mM); Mn^2+^ (0.1 mM)

All fractions of both the sets were incubated for 2 h at 37°C and evaluated for the following parameters:

### Determination of thiols

TSH, GSH, GSSG and redox ratio (GSH/GSSG) were determined by the standard methods.[19]

### Estimation of total thiols

The assay mixture containing 0.2 M Tris-buffer (pH 8.2) and a known volume of the sperm fraction (control/experimental) was incubated for 30 min at 37°C. 0.1 mL of 0.1 M dithiobis nitrobenzoic acid (DTNB) was added. The volume was raised to 10 mL with absolute methanol and the contents were mixed thoroughly. The capped tubes were allowed to stand at room temperature with occasional shaking for 30 min. The tubes were centrifuged at 2500 ×g for 15 min. The absorbance of the clear aliquot (after filtration through Whatman no. 1) was measured at 412 nm with a spectrophotometer. The molar extinction coefficient (ε) at 412 nm was taken as 131,00/M/cm.

### Estimation of GSH

The assay mixture containing 0.2 M Tris buffer (pH 8.2) and a known volume of the sperm fraction (control/experimental) was incubated for 30 min at 37°C. The volume was raised to 2 mL with 20% chilled trichloro acetic acid. The tubes were vortexed and were allowed to stand, with occasional shaking, for 15 min. The samples were centrifuged at 2500 ×g for 15 min. A 1 mL aliquot was mixed with 2 mL of 0.4 M Tris-buffer (pH 8.9) and 0.1 ml of 0.1 M DTNB. The contents were mixed well and the absorbance was read within 5 min of the addition of DTNB at 412 nm against a reagent blank. The molar extinction coefficient (ε) at 412 nm was 13,100/M/cm.

### Estimation of GSSG

GSSG was calculated by subtracting the GSH from the TSH.

### Redox ratio (GSH/GSSG)

The redox ratio of the FeAA/nicotine or FeAA + nicotine treated/untreated and/or Mn^2+^ supplemented/unsupplemented samples was calculated by dividing the GSH content by the GSSG content of the respective samples.

### Statistics

The data were statistically analyzed by Student “*t*” test. The differences for all the samples from the control (untreated + unsupplemented) were evaluated at the significance levels of *P* < 0.05*, *P* < 0.01** and *P* < 0.001**. A *P*-value < 0.05 was selected as the criterion for statistically significant differences of the samples from the Mn^2+^-supplemented samples, but, given the same treatments.

## RESULTS

### Total thiols

Treatment of the spermatozoal fractions with both the stress inducers (ferrous ascorbate, nicotine) and their combination (ferrous ascorbate + nicotine) gradually increased the TSH content but the increase was significant (*P* < 0.05) with only ferrous ascorbate + nicotine [Table 1]. Supplementation of 0.1 mM Mn^2+^ to the control and stress-induced fractions gradually increased the TSH content, but this increase was significant (*P* < 0.05) only in ferrous ascorbate + nicotine-treated samples [Table 1].

**Table 1:** Total thiol content in the human spermatozoal samples untreated/treated with FeAA, nicotine and FeAA + nicotine and/or unsupplemented/supplemented with Mn^2+^ (0.1 mM)

μ moles -SH/mg prot/min				

Mn^2+^ (mM)	Untreated	Treated		
		
		FeAA	Nicotine	FeAA + Nicotine
0	17.66 ± 1.57 (control)	18.56 ± 1.386	19.85 ± 1.047	21.518 ± 1.06*
0.1	19.78 ± 0.69	21.18 ± 1.27*	19.139 ± 0.59	24.66 ± 1.16*^a^

Each value represents the mean + SE of three independent observations;

a *P* < 0.05 as compared with the Mn^2+^-supplemented samples treated similarly;

* *P* < 0.05 as compared with the control samples

### Glutathione oxidized

GSH content of human spermatozoa decreased nonsignificantly on treating with both the stress inducers, ferrous ascorbate, nicotine and their combination (ferrous ascorbate + nicotine) [Table 2]. Mn^2+^ supplementation significantly increased the TSH content in all the stress-induced fractions except in the control [Table 2].

**Table 2:** Glutathione reduced (GSH) content in the human spermatozoal samples untreated/treated with FeAA, nicotine and FeAA + nicotine and/or unsupplemented/supplemented with Mn^2+^ (0.1 mM)

μ moles -SH/mg prot/min				

Mn^2+^ (mM)	Untreated	Treated		
		
		FeAA	Nicotine	FeAA + Nicotine
0	13.28 ± 0.464 (control)	12.49 ± 0.54	12.81 ± 0.40	11.20 ± 0.84
0.1	13.48 ± 0.67	16.53 ± 0.50^b^	15.07 ± 0.77^a^	18.38 ± 0.623^c^*

Each value represents the mean + SE of three independent observations;

a *P* < 0.05 as compared with the Mn^2+^-supplemented samples treated similarly;

* *P* < 0.05 as compared with the control samples

### Glutathione oxidized and redox ratio

Treatment of the spermatozoa with both the stress inducers ferrous ascorbate, nicotine and their combination (ferrous ascorbate + nicotine) significantly (*P* < 0.01) increased the GSSG level [Table 3]. Mn^2+^ supplementation decreased the GSSG level in all the stress-induced fractions, but significantly (*P* < 0.05) in the nicotine and ferrous ascorbate + nicotine-treated fractions [Table 3].

**Table 3:** Glutathione oxidized (GSSG) content in the human spermatozoal samples untreated/treated with FeAA, nicotine and FeAA + nicotine and/or unsupplemented/supplemented with Mn^2+^ (0.1 mM)

μ moles -SH/mg prot/min				

Mn^2+^ (mM)	Untreated	Treated		
		
		FeAA	Nicotine	FeAA + Nicotine
0	4.38 ± 0.380 (control)	6.06 ± 0.221**	7.03 ± 0.584**	10.309 ± 1.080**
0.1	1.296 ± 0.180***	4.657 ± 0.77	4.068 ± 1.289^a^	6.287 ± 0.962^a^

Each value represents the mean + SE of three independent observations;

a *P* < 0.05 as compared with the Mn^2+^-supplemented samples treated similarly;

* *P* < 0.05 as compared with the control samples

### Redox ratio (GSH/GSSG)

A gradual decrease in the redox ratio was observed by treating the sperm fractions with both the stress inducers and their combination [Table 4]. But, supplementation of Mn^2+^ to the control as well as the stress-induced fractions increased the redox ratio [Table 4].

**Table 4:** Redox ratio (GSH/GSSG) in the human spermatozoal samples untreated/treated with FeAA, nicotine and FeAA + nicotine and/or unsupplemented/supplemented with Mn^2+^ (0.1 mM)

μ moles -SH/mg prot/min				

Mn^2+^ (mM)	Untreated	Treated		
		
		FeAA	Nicotine	FeAA + Nicotine
0(%)	3.02 (100) (control)	2.06 (68.05)	1.82 (60.22)	1.08 (35.91)
0.1(%)	10.40 (343.81)	3.551 (117.31)	3.705 (122.40)	2.92 (96)

Values in parenthesis are percentage, 100% = 3.024 μ moles -SH/mg prot/min

## DISCUSSION

Thiol groups (–SH) are required for reducing the oxidative stress in biological materials.[7] Under oxidative stress conditions (induced by ferrous ascorbate, nicotine, ferrous ascorbate + nicotine) in the human sperm, TSH and GSSG contents increased but the GSH and redox ratio decreased. Subsequently, supplementation of 0.1 mM Mn^2+^ improved the TSH, GSH and redox ratio but decreased the GSSG level. It indicates that Mn^2+^ has been able to maintain the –SH (sulfhydryl group) content under normal and oxidative stress conditions.

TSH is constituted by GSH and GSSG, an increase or decrease of which may affect the level of TSH.[20] In the present study, glutathione acts as an antioxidant in reducing the oxidative stress caused by both the stress inducers (ferrous ascorbate, nicotine) and their combination (ferrous ascorbate + nicotine) in human sperm cells. The antioxidant enzyme, GPx, of this cycle removes peroxyl (ROO) radicals from various peroxides like H_2_O_2_ and, thus, converts GSH to GSSG, whereas GR regenerates GSH from GSSG, as shown below[3]:

$\begin{array}{l} 2{\text{GSH+H}}_{2}{\text{O}}_{2}\overset{\text{GPx}}{\to }{\text{GSSH+H}}_{2}\text{O}\\ {\text{GSSH+NADP+H}}^{+}\overset{\text{GR}}{\to }2{\text{GSH+NADP}}^{+} \end{array}$

Data analysis shows that both the stress inducers (ferrous ascorbate, nicotine) and their combination (ferrous ascorbate + nicotine) decrease the GSH content but increase the TSH and GSSG levels. However, significant differences are observed in the samples treated with the combination of the stress inducers. This may be due to the reason that the combination of ferrous ascorbate and nicotine induces maximum oxidative stress in sperm cells and, to overcome this stress, GSH gets converted to GSSG through the reaction catalyzed by GPx (antioxidant enzyme of glutathione cycle). The decrease in the GSH content during aerobic incubation could be due to the transport of this molecule out of the sperm cell.[11] The decrease in GSH and the increase in TSH and GSSG contents induced by both the stress inducers and their combination in human sperm may be to overcome the oxidative damages. Thiol groups serve as defense mechanisms of sperm cells to fight against oxidative stress induced by stress inducers such as ferrous ascorbate and nicotine and their combination (ferrous ascorbate 1 nicotine).

Data show that various treatments decrease the redox ratio (GSH/GSSG) gradually. This ratio is inversely related to oxidative stress.[7] To combat the oxidative stress induced by both the stress inducers (ferrous ascorbate, nicotine) and their combination, it is suggested to decrease content but increase the GSSG level, thereby resulting in the decrease of the GSH/GSSG ratio.

Data analysis shows that Mn^2+^ supplementation to the treated (ferrous ascorbate, nicotine, ferrous ascorbate + nicotine) as well as control samples increased the TSH and GSH contents but decreased the GSSG level. This may be due to the anti-oxidative nature of Mn^2+^, which increases the GSH and TSH contents but decreases the GSSG level of the sperm membranes. It is suggested that Mn^2+^ may stimulate the enzymes of the glutathione cycle and affect the TSH, GSH and GSSG contents. It has been reported that Mn^2+^ decreases the peroxidative damages caused by LPO by conjugating with GPx enzyme in rat brain homogenate. This study shows that with the supplementation of Mn^2+^, oxidative stress decreases significantly under control and treated conditions. Consequently, need of conversion of GSH to GSSG gets lowered. Thus, the level of GSH begins to increase and the GSSG level decreases, thereby maintaining the redox ratio.

## CONCLUSION

Mn^2+^ supplementation maintains thiol levels by reducing oxidative stress.
